# Diabetes Self-Management and Glycemic Control in Adults with Type 2 Diabetes: A Cross-Sectional Analysis with Corrected DSMQ Scoring

**DOI:** 10.3390/diseases14100375

**Published:** 2026-10-12

**Authors:** Paula-Alexandra Popovici, Andreea Diana Igna, Bianca-Lăcrimioara Petca, Corina Cinezan, Timea Claudia Ghitea, Mihaela Simona Popoviciu

**Affiliations:** 1 Doctoral School of Biological and Biomedical Sciences, University of Oradea, 1 University Street, 410087 Oradea, Romania; cipleu.paulaalexandra@student.uoradea.ro (P.-A.P.); igna.andreeadiana@student.uoradea.ro (A.D.I.); criste.biancalacrimioara@student.uoradea.ro (B.-L.P.); 2 Department of Medical Disciplines, Faculty of Medicine and Pharmacy, University of Oradea, 410073 Oradea, Romania; ccinezan@uoradea.ro; 3 Pharmacy Department, Faculty of Medicine and Pharmacy, University of Oradea, 1 University Street, 410087 Oradea, Romania; 4 Department of Preclinical Disciplines, Faculty of Medicine and Pharmacy, University of Oradea, 410028 Oradea, Romania; mihaela.popoviciu@didactic.uoradea.ro

**Keywords:** type 2 diabetes, diabetes self-management, DSMQ, HbA1c, cross-sectional study, data quality, patient-reported outcome

## Abstract

Background: Accurate scoring is essential when patient-reported self-management instruments are related to biochemical outcomes. The principal contribution of this study was to reconstruct and audit Diabetes Self-Management Questionnaire (DSMQ) scores from item-level data before reassessing their cross-sectional association with glycemic control. Methods: We performed a cross-sectional secondary analysis of a clinical database of adults with type 2 diabetes. One non-patient header row was removed. Because 12 observation-sheet identifiers occurred twice and visit dates were unavailable, all 24 records carrying repeated identifiers were excluded from the prespecified primary analysis, leaving 238 records. Negatively keyed DSMQ items were reverse-scored, and total and subscale scores were transformed to a 0–10 scale according to the original algorithm. Associations with HbA1c were assessed using Spearman correlation, exploratory ROC analysis, and multivariable linear regression with HC3 robust standard errors. Results: HbA1c was available for 237 records; mean age was 64.18 years (SD 11.14), 56.1% of records with valid sex coding were female, and mean HbA1c was 7.81% (SD 1.94). The corrected DSMQ total score was 5.19 (SD 2.36). DSMQ total score correlated inversely with HbA1c (ρ = −0.467, bootstrap 95% CI −0.572 to −0.350; *p* < 0.001). In the adjusted model (*n* = 236), each one-point higher DSMQ total score was associated with a 0.254 percentage-point lower HbA1c (regression coefficient = −0.254, 95% CI −0.344 to −0.163; *p* < 0.001). Sensitivity analyses using all records or first/last repeated-record rules produced similar estimates. Conclusions: Correctly scored DSMQ results showed a moderate, independent cross-sectional association with HbA1c. The corrected analysis did not demonstrate excellent discrimination, and the cross-sectional data cannot support causal or predictive claims. Prospective studies with verified unique participants and clearly documented data provenance are needed.

## 1. Introduction

Type 2 diabetes requires sustained daily self-management, including medication use, glucose monitoring when indicated, dietary adjustment, physical activity, and effective interaction with the health-care team. Contemporary diabetes guidance emphasizes individualized glycemic goals and structured diabetes self-management education and support as core components of care [1,2].

The 16-item Diabetes Self-Management Questionnaire (DSMQ) was developed to assess self-care behaviors that are relevant to glycemic control. It yields a total score and four subscales: Glucose Management, Dietary Control, Physical Activity, and Health-Care Use. The original validation study reported a correlation of approximately −0.40 between the DSMQ total score and HbA1c, and subsequent studies have supported its utility across languages and clinical populations [3,4,5,6,7].

Although previous studies have reported significant relationships between DSMQ scores and HbA1c, the existence of an association does not remove the need for a reproducible reanalysis. The present study addresses a different question from simple replication: whether the association remains after item-level reconstruction of the DSMQ, explicit handling of repeated identifiers and implausible laboratory entries, recalculation of BMI, and prespecified sensitivity analyses. It also evaluates whether the corrected score adds information after adjustment for clinical characteristics and whether its apparent discrimination is sufficiently strong to justify threshold-based or predictive interpretations.

Furthermore, diabetes self-management is influenced by multiple demographic and clinical factors, including age, duration of diabetes, obesity, treatment intensity, educational level, and health-care accessibility. These factors may modify the relationship between self-management behaviors and metabolic outcomes, emphasizing the need for multivariable analyses capable of distinguishing behavioral effects from underlying disease severity [8,9].

Because the DSMQ includes nine negatively keyed items, incorrect reverse scoring can materially alter total and subscale values. Data-derived thresholds can also appear overly accurate when developed and evaluated in the same sample. Therefore, the present analysis aimed to reassess the association between DSMQ scores and glycemic outcomes using item-level recalculation, explicit data-quality rules, robust regression, and sensitivity analyses.

## 2. Materials and Methods

### 2.1. Study Design and Setting

This cross-sectional study was a secondary analysis of a clinical database containing records of adults with previously diagnosed type 2 diabetes evaluated in the specialized diabetes and nutrition outpatient service of Bihor County Clinical Emergency Hospital, Oradea, Romania, between 1 November 2025 and 30 May 2026. The database included demographic and clinical characteristics, laboratory measurements, treatment information, and item-level responses to the Diabetes Self-Management Questionnaire (DSMQ). The present analysis examined the association between recalculated DSMQ scores and glycemic control. Record selection and data-quality procedures are described in Section 2.2. Recruitment logs and referral information were not available in the analyzed extract; therefore, the numbers screened, excluded before database entry, or declining participation, as well as the referral pathway, could not be reconstructed from the available data.

### 2.2. Eligibility and Data-Quality Rules

Adults aged at least 18 years with previously diagnosed type 2 diabetes and complete responses to all 16 DSMQ items were eligible for analysis. Reasons for non-participation or exclusion before inclusion in the analytical database included refusal to complete the questionnaires, incomplete data, concomitant infections considered capable of affecting laboratory measurements, and withdrawal of informed consent. These recruitment-stage exclusions were distinct from the record-level data-quality procedures described below. The analytical CSV contained 263 rows, including one trailing row containing repeated variable labels rather than patient data. Removal of this non-patient row left 262 patient records corresponding to 250 distinct observation-sheet identifiers. Twelve identifiers appeared twice, accounting for 24 records. Because visit dates and other information needed to distinguish duplicate entries, corrected records, and separate encounters were unavailable, all 24 records bearing repeated identifiers were excluded from the primary analysis, leaving 238 records. Sensitivity analyses applied alternative record-selection rules by retaining all 262 records or retaining only the first or last occurrence of each identifier.

During the initial data screening, one HbA1c entry recorded as 61 and one fasting plasma glucose entry recorded as 2293 mg/dL were treated as missing for analyses involving the corresponding variable. Subsequent verification against the original laboratory records established that the HbA1c value was expressed in IFCC units (61 mmol/mol, equivalent to approximately 7.73% in NGSP units) and confirmed the glucose value as 2293 mg/dL. The analyses currently reported retain the initial treatment of these two entries as missing; therefore, they do not incorporate these subsequently verified measurements. Neither omission resulted in exclusion of the entire patient record. Body mass index was recalculated from recorded weight and height as weight in kilograms divided by height in meters squared. One nonstandard sex code was treated as missing only in analyses requiring sex. No missing values were imputed. Under these analytical rules, the primary cohort comprised 238 records, analyses involving HbA1c or fasting plasma glucose each included 237 records, and the fully adjusted regression models included 236 complete cases.

### 2.3. DSMQ Scoring

The DSMQ was scored from the 16 item responses following the original algorithm [3]. Items 5, 7, and 10–16 were reverse-scored. Using the original 0–3 item coding, each reverse-scored response was transformed as x′i = 3 − xi. For a scale containing k items, the standardized score was calculated as 10 × Σxi/(3k), where xi denotes the scored item response; the total score used k = 16, and each subscale used its prespecified item count. The total score included all 16 items. Glucose Management included items 1, 4, 6, 10, and 12; Dietary Control included items 2, 5, 9, and 13; Physical Activity included items 8, 11, and 15; and Health-Care Use included items 3, 7, and 14. Item 16 contributed only to the total score. Higher scores indicate more effective self-management. An analysis-code or supplementary reproducibility file implementing this formula should accompany the submission so that the corrected scores can be independently reproduced.

### 2.4. Outcomes and Covariates

The primary outcome was HbA1c. Fasting plasma glucose was a secondary outcome. The principal exposure was the corrected DSMQ total score; corrected subscales were examined in secondary analyses. Prespecified regression covariates were age, sex, diabetes duration, recalculated BMI, and basal and/or rapid-acting insulin therapy. The supplied dataset did not contain timestamps for DSMQ completion and laboratory measurement, so their time interval could not be calculated. Insulin therapy was included as an adjustment variable, but because initiation may follow worsening glycemia and may lie on the causal pathway, it is not interpreted as an unambiguous baseline confounder.

### 2.5. Statistical Analysis

Continuous variables are summarized using the mean and standard deviation (SD) and, where informative, the median and interquartile range (IQR). Categorical variables are summarized as counts and percentages. Distributional shape was assessed descriptively using histograms and Q–Q plots; rank-based methods were used when distributions were non-normal or ordinal. DSMQ scores were compared between HbA1c < 7.0% and HbA1c ≥ 7.0% using the Mann–Whitney U test. Associations with HbA1c and fasting plasma glucose were evaluated using Spearman’s rank correlation; 95% confidence intervals (CIs) were obtained with 5000 bootstrap resamples. An exploratory receiver operating characteristic (ROC) analysis estimated the area under the curve (AUC) for discriminating HbA1c < 7.0% from HbA1c ≥ 7.0%; the cutoff was used descriptively and was not proposed as a clinical threshold.

Multivariable ordinary least-squares regression estimated the cross-sectional association with HbA1c using heteroscedasticity-consistent HC3 standard errors. All prespecified predictors were entered simultaneously (enter method). Model 1 included the corrected DSMQ total score and covariates. Model 2 included the four corrected subscales simultaneously with the same covariates. Variance inflation factors assessed multicollinearity. Missing values were not imputed. In the primary cohort, one HbA1c value (1/238; 0.4%) and one sex value (1/238; 0.4%) were missing; the other model variables and DSMQ scores were complete. Regression therefore used complete cases (*n* = 236), and the missing-data mechanism was not formally assessed because of the very small number of missing observations. No formal a priori sample-size calculation was available for this secondary analysis; the sample size was determined by the source database, so imprecision and Type II error remain possible. Analyses were performed using Python 3.11.15, pandas 2.2.3, SciPy 1.15.2, statsmodels 0.14.4, and scikit-learn 1.5.2.

### 2.6. Ethics

The supplied documentation identifies approval by the Ethical Committee of the Faculty of Medicine and Pharmacy, University of Oradea (approval no. 41, 31 October 2025) and written informed consent from participants. The original study/database name and protocol should be stated explicitly here rather than referring vaguely to a “source manuscript”.

## 3. Results

### 3.1. Cohort and Data Integrity

The source file contained 263 rows. After removal of one non-patient header row, 262 patient rows remained; conservative exclusion of the 24 records associated with 12 repeated identifiers left 238 records in the primary analysis. HbA1c was evaluable in 237 records, and the fully adjusted models included 236 complete cases. No screening or declination log was supplied, so the numbers screened, eligible, excluded, and declining participation cannot be reported from the extract alone.

Recalculation identified disagreement between the stored and corrected DSMQ total score in 249 of 262 patient rows. The stored BMI differed from BMI recalculated using weight and height by more than 0.1 kg/m^2^ in 28 rows and by more than 1.0 kg/m^2^ in nine rows.

### 3.2. Participant Characteristics

The conservative primary analysis cohort comprised 238 patients. The mean age was 64.18 years (SD 11.14), and women represented 56.1% of participants with available sex data. More than half of the cohort lived in rural areas (55.9%). The median duration of diabetes was 7.00 years (IQR 4.00 to 14.00), while the mean recalculated BMI was 31.75 kg/m^2^ (SD 5.94), indicating that obesity was common in this population.

After retaining the prespecified missing-data treatment for the verified laboratory entries, the mean HbA1c was 7.81% (SD 1.94), with a median of 7.30% (IQR 6.50 to 8.70). Overall, 96 of 237 patients (40.5%) had an HbA1c below 7.0%. The mean fasting plasma glucose was 153.03 mg/dL (SD 55.48) after retaining the prespecified missing-data treatment for the verified glucose entry.

The mean DSMQ total score was 5.19 (SD 2.36) on the standardized 0–10 scale. Among the DSMQ subscales, Glucose Management (mean 6.15, SD 2.37) and Health-Care Use (mean 6.05, SD 2.57) had the highest mean scores, whereas Dietary Control had the lowest score (mean 4.06, SD 3.21). The mean Physical Activity score was 5.29 (SD 3.39). Finally, 102 patients (42.9%) were receiving basal and/or rapid-acting insulin (Table 1).

### 3.3. Corrected DSMQ Scores and Glycemic Outcomes

The mean corrected DSMQ total score was 5.19 (SD 2.36) on the standardized 0–10 scale. The corrected questionnaire demonstrated high internal consistency, with a Cronbach’s α of 0.917. This estimate describes covariance among the items in this sample; it does not establish unidimensionality, validity, or equivalence with the original instrument.

**Figure 1:**
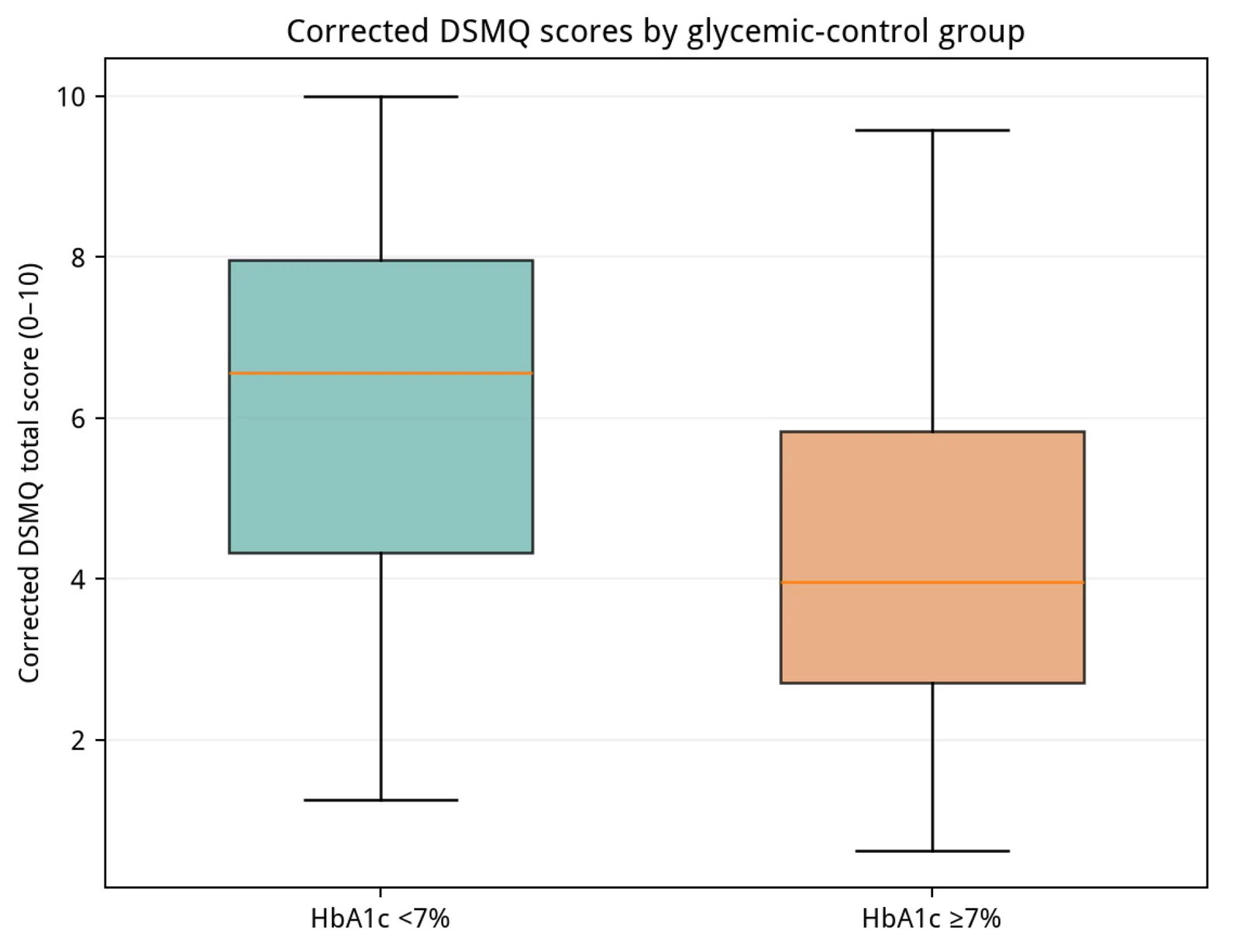
Corrected DSMQ total score according to the conventional HbA1c 7.0% threshold. Individualized glycemic targets were not available.

Participants with HbA1c < 7.0% had substantially higher DSMQ total scores than those with HbA1c ≥ 7.0%. The median score was 6.56 (IQR: 4.32–7.97) in the group below the conventional HbA1c threshold, compared with 3.96 (IQR: 2.71–5.83) in the group with HbA1c ≥ 7.0% (*p* < 0.001). Thus, the median between-group difference was 2.60 points on the 0–10 DSMQ scale. Although some overlap between the score distributions remained, Figure 1 shows a clear upward shift in DSMQ scores among participants with HbA1c < 7.0%.

Spearman correlation analysis further demonstrated that higher self-management scores were consistently associated with lower HbA1c values (Table 2). The corrected DSMQ total score showed a moderate inverse correlation with HbA1c (ρ = −0.467; 95% CI: −0.572 to −0.350; *p* < 0.001). Among the individual DSMQ domains, Glucose Management showed the strongest inverse correlation (ρ = −0.462; 95% CI: −0.568 to −0.349; *p* < 0.001), closely approximating the association observed for the total score.

Health-Care Use and Dietary Control were also moderately inversely correlated with HbA1c, with coefficients of −0.412 (95% CI: −0.520 to −0.293; *p* < 0.001) and −0.402 (95% CI: −0.515 to −0.275; *p* < 0.001), respectively. Physical Activity demonstrated a weaker, although still statistically significant, inverse correlation with HbA1c (ρ = −0.307; 95% CI: −0.425 to −0.185; *p* < 0.001). The confidence intervals for all correlations excluded zero, supporting the consistency of the observed associations across the total score and all four self-management domains.

The scatterplot illustrates the overall inverse relationship between the corrected DSMQ total score and HbA1c. The descriptive LOWESS curve indicates that HbA1c generally declined as the DSMQ score increased. The decrease appeared more pronounced across the lower-to-intermediate DSMQ score range and became more gradual at higher scores. This pattern suggests possible nonlinearity; because no formal spline or polynomial model was prespecified, the primary linear coefficient should be interpreted as an average cross-sectional association rather than a complete description of the curve. Substantial individual variability was present, particularly among participants with lower DSMQ scores, including several markedly elevated HbA1c values.

Collectively, these findings show that higher DSMQ scores were more common among participants with HbA1c below the conventional 7.0% threshold. The Mann–Whitney comparison and Spearman correlation describe group differences and rank association; they do not estimate a likelihood ratio, odds ratio, or probability of achieving the threshold. Because the analyses were cross-sectional and individualized glycemic targets were unavailable, these results should be interpreted as associations rather than evidence of causality or proof that the same HbA1c target was clinically appropriate for every participant (Figure 1 and Figure S1).

### 3.4. Adjusted Association with HbA1c

To examine whether the association between diabetes self-management and glycemic status was independent of measured demographic and clinical characteristics, a multivariable linear regression model was fitted with HbA1c as the dependent variable. The model included the corrected DSMQ total score, age, sex, diabetes duration, recalculated BMI, and treatment with basal and/or rapid-acting insulin. The analysis was based on 236 complete cases: one HbA1c value and one sex value were missing from the 238-person primary cohort, and no imputation was performed. Heteroscedasticity-consistent HC3 standard errors were used to provide more robust statistical inference. Regression coefficients represent adjusted differences in HbA1c percentage points, with negative coefficients indicating lower HbA1c and positive coefficients indicating higher HbA1c. Because insulin treatment may be initiated after worsening glycemia, its coefficient is interpreted cautiously and not as evidence of a causal effect of insulin (Table 3).

After adjustment, each one-point higher DSMQ total score was associated with a 0.254 percentage-point lower HbA1c (95% CI −0.344 to −0.163; *p* < 0.001). Longer diabetes duration and insulin treatment were associated with a higher HbA1c, whereas BMI was not associated with HbA1c in this model.

In the secondary model containing all four subscales, only Glucose Management retained an inverse association with HbA1c after simultaneous adjustment (regression coefficient = −0.180, 95% CI −0.320 to −0.041; *p* = 0.011). Dietary Control (*p* = 0.088), Physical Activity (*p* = 0.260), and Health-Care Use (*p* = 0.308) were not independently associated after shared variance was removed. This pattern should not be interpreted as evidence that the other domains are clinically unimportant; simultaneous adjustment estimates domain-specific coefficients in the presence of correlated components of the same instrument. All variance inflation factors were below 3.30.

### 3.5. Sensitivity Analyses

Sensitivity analyses examined whether alternative rules for repeated identifiers changed the corrected DSMQ–HbA1c association. Across inclusion of all records and retention of the first or last repeated-ID record, the Spearman coefficients ranged from −0.464 to −0.488, and adjusted regression coefficients from −0.247 to −0.265 HbA1c percentage points per one-point DSMQ increase (Supplementary Table S1). Because these estimates were closely similar, detailed duplicate-handling results are provided as Supplementary Information rather than emphasized in the main text.

The inverse correlation between the corrected DSMQ total score and HbA1c remained consistent across all cohort definitions, with Spearman coefficients ranging from −0.464 to −0.488. Similarly, the adjusted regression coefficient ranged from −0.247 to −0.265 HbA1c percentage points per one-point increase in the DSMQ score; all corresponding CIs remained below zero. These analyses address uncertainty related to repeated identifiers but do not eliminate the limitations inherent to the cross-sectional observational design.

In the conservative primary cohort, each one-point increase in the DSMQ total score was associated with a 0.254-percentage-point lower HbA1c. The estimated association was similar when all 262 records were included (coefficient = −0.258), when the first record for each repeated identifier was retained (coefficient = −0.247), and when the last record was retained (coefficient = −0.265).

These results indicate that neither the direction nor the magnitude of the association changed materially under the alternative duplicate-record rules. They support the robustness of the primary estimate, but they cannot determine whether repeated identifiers represented duplicate entries, corrected forms, or genuine repeat visits; source-document adjudication remains preferable.

## 4. Discussion

The present study shows that, after complete item-level rescoring of the 16-item DSMQ and conservative management of repeated identifiers, the corrected score had a moderate inverse cross-sectional association with HbA1c. The central novelty is methodological: reconstruction of the score from item-level data and transparent correction of database-quality problems, rather than a new claim that an association between self-management and glycemia exists. If this manuscript is intended as a correction or reanalysis of a previous report, the publication making the original claims of a very strong correlation, excellent discrimination, or predictive/causal effects must be identified explicitly here [10,11]. In the absence of that citation, those claims should not be attributed to a prior publication.

The magnitude of the corrected correlation is clinically and psychometrically plausible. It is close to the approximately −0.40 association reported in the original DSMQ validation study [3], while the present estimate was −0.467. Subsequent linguistic and clinical validation studies have reported heterogeneous associations [4,5,6,7], and direct head-to-head comparison is limited by differences in population, treatment setting, outcome timing, and scoring implementation. This convergence supports the plausibility of the corrected scores without establishing equivalence across instruments or populations.

The high internal consistency observed in this cohort (Cronbach’s alpha = 0.917) indicates strong covariance among the DSMQ items. This value supports score reliability in the analyzed sample, but it should not be interpreted as proof that the questionnaire is unidimensional or that all items measure an identical behavioral construct. The DSMQ was intentionally designed to capture several related components of self-management, and a very high alpha may partly reflect overlap among behaviors that tend to occur together. Accordingly, internal consistency should be considered alongside the established domain structure and the observed relationships with HbA1c rather than used as a stand-alone indicator of validity [12,13,14].

The adjusted effect estimate provides a more clinically interpretable expression of the association. Under the fitted linear model, a three-point difference in DSMQ total score corresponds to an estimated difference of approximately 0.76 percentage points in HbA1c among participants with otherwise similar measured covariates. This calculation should be viewed as an adjusted cross-sectional contrast and not as the expected effect of increasing an individual’s DSMQ score through an intervention. HbA1c reflects glycemia over the preceding months, whereas the DSMQ captures recently reported behaviors; without repeated measurements, the temporal order between behavior and glycemic status cannot be established. Reverse causation is also possible because awareness of poor glycemic control may prompt patients to intensify self-care or, conversely, may contribute to discouragement and lower reported adherence [15].

Among the four DSMQ domains, Glucose Management showed the strongest unadjusted relationship with HbA1c and was the only domain that retained an independent inverse association when all subscales were entered simultaneously. This pattern is biologically and behaviorally credible because medication-taking, glucose monitoring, and responses to abnormal glucose values are more proximal to measured glycemia than many other self-care behaviors [3,16,17]. Nevertheless, the finding should not be interpreted as evidence that Glucose Management is the only clinically relevant domain. Dietary Control, Physical Activity, and Health-Care Use were each inversely correlated with HbA1c in unadjusted analyses, but their coefficients were attenuated after mutual adjustment. Because self-management behaviors cluster within individuals, simultaneous inclusion of correlated subscales estimates the unique contribution of each domain after removing their shared variance. This analytical approach may therefore underestimate the broader contribution of mutually reinforcing behaviors [4,18,19,20].

Dietary Control had the lowest mean score in the cohort, suggesting a potentially important area of unmet support. Its lack of statistical independence in the simultaneous subscale model does not exclude clinical relevance. Dietary behaviors can influence postprandial glucose, body weight, lipid profiles, and treatment requirements through pathways that may not be fully represented by a single HbA1c measurement. Similarly, the weaker association for Physical Activity may reflect measurement limitations, differences in exercise intensity and duration, mobility restrictions in an older population, or indirect metabolic effects. Health-Care Use may also show a complex relationship with HbA1c because more frequent contact can represent either proactive care or a response to difficult-to-control diabetes. These possibilities emphasize the difference between absence of an independent coefficient and absence of clinical importance [21,22,23,24].

Longer diabetes duration and treatment with basal and/or rapid-acting insulin were independently associated with higher HbA1c. These findings are compatible with increasing beta-cell dysfunction, treatment complexity, and disease severity over time. The positive coefficient for insulin treatment should not be interpreted as evidence that insulin worsens glycemic control: insulin may have been initiated in response to prior hyperglycemia and may therefore lie on the causal pathway or act as a marker of disease severity. Because this study is cross-sectional, the temporal ordering among diabetes duration, HbA1c, DSMQ score, and insulin treatment cannot be established.

The corrected DSMQ total score showed only moderate apparent discrimination between participants below and above the conventional HbA1c threshold (AUC = 0.730; bootstrap 95% CI 0.661 to 0.793). This ROC result is reported here for completeness and should be viewed as exploratory, internally estimated performance rather than validated diagnostic or prognostic accuracy. It is materially more realistic than an excellent classification claim and is consistent with the multifactorial nature of glycemic control. Because the <7.0% threshold is not appropriate for every adult with type 2 diabetes and individualized targets were unavailable, the present study does not propose a clinical DSMQ cutoff.

### 4.1. Clinical Implications

The DSMQ may be useful as a complementary patient-reported measure for identifying behavioral domains that warrant further discussion during routine diabetes care. A low total score should not be treated as a diagnosis of nonadherence, and a high score should not be assumed to guarantee adequate glycemic control. Instead, the questionnaire can provide a structured starting point for shared decision-making, allowing clinicians to explore medication-taking difficulties, glucose-monitoring practices, dietary barriers, physical limitations, diabetes distress, health literacy, and access to professional support. The domain profile may be more actionable than the total score alone because two patients with similar total scores can have different self-management needs [25].

The results also support the continued integration of structured diabetes self-management education and support into clinical care, as recommended by current guidance [1,2]. However, the present study did not evaluate an educational intervention and cannot quantify the HbA1c reduction that would follow from improving a DSMQ score. Prospective studies should determine whether changes in DSMQ scores track changes in HbA1c over time and whether questionnaire-guided interventions improve outcomes beyond usual care. For clinical implementation, DSMQ results should be interpreted together with biochemical monitoring, medication history, hypoglycemia risk, comorbidities, functional status, and individualized treatment goals [26].

### 4.2. Data-Quality and Methodological Implications

A central contribution of this analysis is methodological. Reconstructing scale scores from item-level responses, verifying the direction of negatively keyed items, recalculating derived anthropometric measures, and examining duplicate identifiers materially changed the results. These steps are especially important when patient-reported instruments are linked to objective clinical outcomes because a scoring error can create apparently precise but invalid associations. Future studies using DSMQ data should retain the item-level dataset, document the scoring syntax, verify theoretical score ranges, inspect agreement between stored and recalculated variables, and establish a reproducible rule for repeated visits and duplicate records before inferential analysis [27,28,29].

The sensitivity analyses provide reassurance regarding the repeated-identifier problem: the adjusted DSMQ coefficient varied only from −0.247 to −0.265 across the four cohort rules. Nevertheless, statistical stability cannot determine whether the repeated identifiers represented duplicate data entries, corrected forms, or genuine repeat visits. Source-document adjudication remains preferable. If repeated observations are confirmed as longitudinal visits, they should not be treated as independent records; analyses would instead require explicit time ordering and methods that account for within-patient correlation [30].

### 4.3. Strengths and Limitations

The principal strengths of this study include item-level reconstruction of the DSMQ total and subscale scores according to the original algorithm, transparent data-quality rules, recalculation of BMI from its source variables, use of bootstrap confidence intervals for rank correlations, and HC3 heteroscedasticity-consistent standard errors in multivariable regression. The conservative primary cohort reduced the risk of counting the same individual twice, while alternative duplicate-handling rules demonstrated that the main association was robust. The simultaneous subscale model and assessment of variance inflation also allowed a more cautious evaluation of the independent information contributed by each DSMQ domain.

Several limitations require consideration. First, the cross-sectional design precludes conclusions about temporality, causality, prediction, or responsiveness to intervention; the interval between DSMQ completion and HbA1c measurement was unavailable. Second, this was a single-center analysis of patients attending a specialized outpatient service, which may limit generalizability to primary care, hospital-based populations, other regions, or patients with limited access to diabetes services. Third, self-management was self-reported and may be affected by recall error and social-desirability bias. Fourth, residual confounding is likely because the available data did not include educational attainment, socioeconomic status, health literacy, diabetes distress, depression, cognitive status, comorbidity burden, detailed medication classes, treatment adherence, dietary intake, or objective physical-activity measures. Fifth, no formal a priori sample-size calculation was available for this secondary analysis, so non-significant results may reflect limited power (Type II error). Sixth, the number of statistical tests and multiple reported *p* values create a possibility of Type I error inflation; the findings should therefore be interpreted as estimation of prespecified associations rather than as proof that every individual test is independently confirmatory. Seventh, repeated identifiers could not be adjudicated because visit dates and source-record information were unavailable. Eighth, one nonstandard sex code and two laboratory entries were initially treated as missing despite subsequent source-record verification, and missing data were analyzed without imputation. Finally, the discrimination analysis was internal and exploratory, with no independent validation sample.

Future research should use prospectively verified unique participants, record the temporal relationship between DSMQ completion and laboratory measurement, and incorporate repeated DSMQ and HbA1c assessments. Multicenter recruitment would improve external validity, while prespecified models should include psychosocial, socioeconomic, treatment, and complication-related factors. Longitudinal analyses could assess whether within-person changes in DSMQ scores precede changes in glycemic outcomes, and randomized studies could test whether domain-targeted support based on DSMQ profiles provides benefit beyond standard diabetes education. External validation would be required before using the questionnaire for classification or applying a numerical threshold in clinical practice.

## 5. Conclusions

Correctly scored DSMQ results showed a moderate inverse cross-sectional association with HbA1c in adults with type 2 diabetes. The total score remained associated with HbA1c after adjustment, and Glucose Management showed the strongest domain-specific association in the simultaneous subscale model; this should not be interpreted as evidence that the other domains lack clinical importance. The principal contribution is a reproducible correction of item-level DSMQ scoring and transparent handling of duplicate identifiers and implausible laboratory entries. The supplied database does not support causal or predictive conclusions, and any comparison with previously reported claims requires explicit citation of the original publication. Future prospective analyses should use verified unique participants, prespecified outcomes, individualized glycemic targets where possible, documented timing of DSMQ and HbA1c measurements, and external validation.

## Figures and Tables

**Table 1:** Characteristics of the conservative primary analysis cohort. Continuous variables are reported as mean (SD) or median (IQR); categorical variables are reported as N/N (%), with percentages rounded to whole numbers. BMI was recalculated from weight and height. HbA1c and fasting glucose each exclude one verified laboratory entry that was retained as missing according to the prespecified analytical rule. Abbreviations: BMI, body mass index; DSMQ, Diabetes Self-Management Questionnaire; HbA1c, glycated hemoglobin; IQR, interquartile range; SD, standard deviation.

Variable (Available *n*)	Mean (SD) or Median (IQR)	N/N (%)
Continuous variables		
Analysis-ready records (*n* = 238)	238	
Age, years (*n* = 238)	64.18 (11.14)	
Diabetes duration, years (*n* = 238)	7.00 (4.00 to 14.00)	
Recalculated BMI, kg/m^2^ (*n* = 238)	31.75 (5.94)	
HbA1c, % (*n* = 237)	7.81 (1.94)	
HbA1c, median (IQR), % (*n* = 237)	7.30 (6.50 to 8.70)	
Fasting plasma glucose, mg/dL (*n* = 237)	153.03 (55.48)	
DSMQ total score, 0–10 (*n* = 238)	5.19 (2.36)	
Glucose Management, 0–10 (*n* = 238)	6.15 (2.37)	
Dietary Control, 0–10 (*n* = 238)	4.06 (3.21)	
Physical Activity, 0–10 (*n* = 238)	5.29 (3.39)	
Health-Care Use, 0–10 (*n* = 238)	6.05 (2.57)	
Categorical variables		
Female sex (*n* = 237)		133/237 (56)
Male sex (*n* = 237)		104/237 (44)
Rural residence (*n* = 238)		133/238 (56)
HbA1c < 7.0% (*n* = 237)		96/237 (41)
Basal and/or rapid-acting insulin (*n* = 238)		102/238 (43)

**Table 2:** Spearman correlations between correctly scored DSMQ measures and HbA1c. Confidence intervals were estimated using 5000 bootstrap resamples. Abbreviations: CI, confidence interval; DC, Dietary Control; DSMQ, Diabetes Self-Management Questionnaire; GM, Glucose Management; HU, Health-Care Use; PA, Physical Activity.

DSMQ Measure	*N*	Spearman ρ	95% CI	*p*
DSMQ total	237	−0.467	−0.572 to −0.350	<0.001
GM	237	−0.462	−0.568 to −0.349	<0.001
DC	237	−0.402	−0.515 to −0.275	<0.001
PA	237	−0.307	−0.425 to −0.185	<0.001
HU	237	−0.412	−0.520 to −0.293	<0.001

**Table 3:** Multivariable linear regression for HbA1c (*n* = 236). R^2^ = 0.378; adjusted R^2^ = 0.361. The regression coefficient is the adjusted difference in HbA1c percentage points per unit increase in the predictor, shown in the same cell as the 95% CI. HC3 heteroscedasticity-consistent standard errors were used. Reference categories were male sex and no basal or rapid-acting insulin treatment. Abbreviations: BMI, body mass index; CI, confidence interval; DSMQ, Diabetes Self-Management Questionnaire; HbA1c, glycated hemoglobin; HC3, heteroscedasticity-consistent covariance estimator 3.

Variable	Regression Coefficient (95% CI)	*p*
DSMQ total, per 1 point	−0.254 (−0.344 to −0.163)	<0.001
Age, per year	−0.031 (−0.055 to −0.007)	0.012
Female sex	0.342 (−0.048 to 0.732)	0.086
Diabetes duration, per year	0.101 (0.060 to 0.143)	<0.001
BMI, per kg/m^2^	−0.001 (−0.036 to 0.033)	0.942
Basal and/or rapid-acting insulin	0.620 (0.108 to 1.131)	0.018

## Data Availability

The original contributions presented in this study are included in this article. Further inquiries can be directed to the first authors.

## Supplementary Materials

The following supporting information can be downloaded at https://www.mdpi.com/article/10.3390/diseases14100375/s1, Table S1: Sensitivity analyses for duplicate-identifier handling. Figure S1. Association between corrected DSMQ total score and HbA1c.

## Abbreviations

The following abbreviations are used in this manuscript:

AUC	Area under the receiver operating characteristic curve
BMI	Body mass index
CI	Confidence interval
DSMQ	Diabetes Self-Management Questionnaire
HbA1c	Glycated hemoglobin
IQR	Interquartile range
OR	Odds ratio
ROC	Receiver operating characteristic
SD	Standard deviation
T2DM	Type 2 diabetes mellitus
