# Supplementary material for: Diabetes Self-Management and Glycemic Control in Adults with Type 2 Diabetes: A Cross-Sectional Analysis with Corrected DSMQ Scoring
Source: Diseases. 2026 Oct 12;14(10):375. doi: 10.3390/diseases14100375 (PMC13703674; doi:10.3390/diseases14100375)
Supplement: Supplementary file 1 [file diseases-14-00375-s001.zip › diseases-4580566-supplementary.pdf]

Supplementary Table S1. Sensitivity analyses for duplicate-identifier handling. The direction and magnitude of the corrected association were stable.

Cohort rule	Rows	Spearman ϱ	Adjusted regression coefficient	95% CI
All 262 records	262	-0.484	-0.258	-0.344 to -0.173
Primary: exclude all repeated IDs	238	-0.467	-0.254	-0.344 to -0.163
Keep first repeated-ID record	250	-0.464	-0.247	-0.336 to -0.158
Keep last repeated-ID record	250	-0.488	-0.265	-0.352 to -0.178

Abbreviations: CI, confidence interval; DSMQ, Diabetes Self-Management Questionnaire; ϱ, Spearman correlation coefficient.

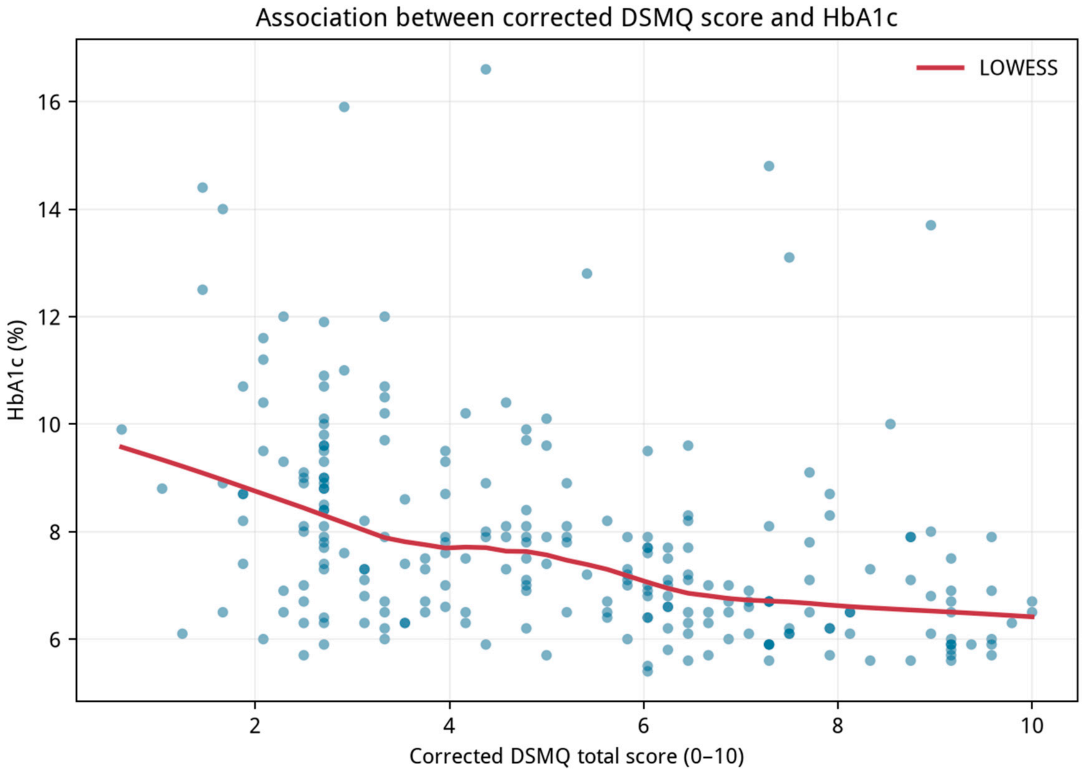

Supplementary Figure S1. Association between corrected DSMQ total score and HbA1c. The red line is a LOWESS smoother and is descriptive, not a causal model.
